# Low metformin concentrations in obese people with HIV treated with dolutegravir

**DOI:** 10.4102/phcfm.v17i1.5096

**Published:** 2025-09-11

**Authors:** Roland van Rensburg, Eric H. Decloedt

**Affiliations:** 1 Division of Clinical Pharmacology, Department of Medicine, Faculty of Medicine and Health Sciences, Stellenbosch University, Cape Town, South Africa

## Introduction

With the success of antiretroviral therapy (ART), obesity and metabolic diseases are emerging concerns in people with HIV (PWH), especially in sub-Saharan Africa, where nearly a third are overweight or obese. Obesity disproportionately affects black African women with HIV, increasing their risk of dysglycaemia. Metformin, the cornerstone of dysglycaemia treatment, is often co-administered with the globally-recommended first-line ART, dolutegravir (DTG). However, the guideline recommendation limiting metformin to 1000 mg/day when used with DTG was based on data from non-obese healthy volunteers, who were mostly white and male. Primary healthcare clinicians also often struggle to control dysglycaemia in PWH with the metformin dose limitation. A study evaluated the pharmacokinetics of metformin and DTG in the target population of 15 obese black African women with HIV, finding markedly lower metformin exposures than previously reported. These findings led to the updated national South African Standard Treatment Guidelines, allowing metformin dosing up to 2000 mg/day with DTG.

## The growing problem of obesity in people with HIV

In the era of potent antiretroviral therapy (ART), people with HIV (PWH) are experiencing near-normal life expectancy.^1^ However, this success has unveiled new challenges, such as the rising tide of non-communicable diseases, including obesity and metabolic disease. In sub-Saharan Africa (SSA), where the majority of the global HIV burden resides, approximately one in three PWH are overweight or obese (body mass index [BMI] ≥25 kg/m^2^).^2,3^ Among women with HIV (WWH) – who constitute nearly two-thirds of PWH in SSA – obesity rates approach 24%, almost double those of men.^4^ Black African women are disproportionately affected, with pooled trial data showing that black WWH on ART gained an additional 2.7 kg over 96 weeks compared with their non-black counterparts.^5^

This weight gain carries substantive health risks. In PWH, each 2.3 kg of weight gain translates into a 14% higher risk of developing type 2 diabetes mellitus (T2DM), versus an 8% increase in HIV-negative populations.^6^ As a result, about one-third of PWH develop dysglycaemia – either overt T2DM or pre-diabetes – necessitating glucose-lowering therapy.^7^ Unfortunately, real-world data from South Africa reveal that overall glycaemic control in diabetic patients is poor, but that control in diabetic PWH is much lower than in people with diabetes alone: only around 15% of diabetic PWH achieve glycated haemoglobin (HbA1c) targets, compared to 25% of HIV-negative controls.^8,9^

## The current evidence of the metformin–dolutegravir drug interaction

Globally, the first-line oral drug for dysglycaemia is metformin, as it is affordable, safe, and promotes weight neutrality or modest weight loss.^10^ With SSA’s expanding ART programmes, concomitant prescription of metformin and the global first-line ART – integrase strand transfer inhibitors, such as dolutegravir (DTG) – is becoming more common.^11^ In South Africa alone, over 1–1.5 million PWH are estimated to receive both drugs daily.^12,13^

A 2016 study by Song et al. identified a significant pharmacokinetic (PK) interaction between metformin and DTG.^14^ When metformin 1000 mg/day was co-administered with DTG 50 mg once daily, metformin’s area under the concentration-time curve (AUC) increased by 79%. The mechanism is via DTG’s inhibition of renal organic cation transporter 2 (OCT2), which facilitates metformin’s exclusive renal elimination. Inhibiting this transporter reduces metformin clearance, increasing plasma concentrations.^15^

On the strength of this single study, major clinical guidelines^16^ and drug interaction databases^17^ recommended limiting metformin to 1000 mg/day when co-administered with DTG 50 mg/day. This recommendation confines metformin to the lower range of dosing, as it can be titrated up to 3000 mg/day. However, the drug interaction study was conducted in 15 healthy volunteers, who were mostly white (93%), male (73%), and – importantly – with a non-obese BMI.^14^ The studied population excluded the target population of PWH in SSA, who are mostly black African (97%),^18^ female (64%),^19^ and more than a quarter being obese with a BMI of >30 kg/m^2^.^4^

## Challenging the status quo

**FIGURE 1:**
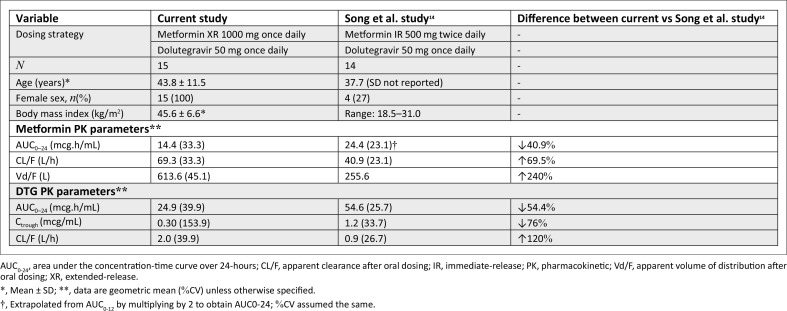
Comparative pharmacokinetic parameters.

To investigate whether the Song findings hold true in the target population, we conducted an observational PK study in 15 black African WWH with class II–III obesity (mean BMI 45.6 kg/m^2^) on DTG and tenofovir disoproxil fumarate and lamivudine and metformin extended-release (XR) 1000 mg once daily.^20^ Intensive plasma sampling over eight timepoints (pre-dose to 12 h post-dose) under standardised meal conditions yielded striking results (Figure 1): we found that the metformin AUC over 24-h (AUC_0–24_) was 40.9% lower than what was reported by Song. The DTG AUC_0–24_ was 54.4% lower compared to the findings of Song.

These findings directly challenge the rationale for a universal metformin cap of 1000 mg/day in obese PWH. The mechanism behind the lower metformin and DTG exposures is likely due to obesity inducing physiological changes that alter drug pharmacokinetics:^21^

- **Increased clearance and volume of distribution:** Body weight is strongly correlated with increased metformin and DTG clearance and expanded adipose tissue sequesters metformin, raising the volume of distribution.^22,23,24^
- **Attenuated transporter inhibition:** Lower DTG plasma concentrations reduce its inhibitory effect on renal OCT2 transporters, increasing metformin clearance.

In short, obese PWH experience both enhanced elimination of metformin and diminished DTG-mediated transporter blockade – netting lower metformin exposure despite the nominal drug interaction. As a result, obese PWH are likely being underdosed with metformin, despite the wide therapeutic range and established safety profile. Indeed, the clinical relevance of this interaction has been questioned by several others, in particular those in primary care.^25,26,27^

## The way forward

Given metformin’s broad safety margin and critical role in weight management and glycaemic control, capping the dose at 1000 mg/day in obese PWH may inadvertently compromise diabetes outcomes. Based on our findings, the South African National Department of Health’s Standard Treatment Guidelines have been updated to increase the maximum metformin dose to 2000 mg/day when co-administered with DTG 50 mg/day, irrespective of BMI.^28^ One of the most reputable ART drug interaction tools, the Liverpool HIV Drug Interactions checker, has also been updated with our data.^29^

## Conclusion

Our data showed that the uniform metformin dose restriction is inappropriate for PWH on DTG, given metformin’s wide therapeutic index and established safety profile. Our data have led to the revision of the maximum metformin dose to 2000 mg/day when co-administered with DTG 50 mg/day, as amended in the updated South African National Department of Health’s Standard Treatment Guidelines. Future studies should focus on incorporating a wider range of body mass indices and modelling approaches to establish the optimal metformin dose in obese PWH on DTG.
